# Neurofeedback Training for Managing Neuropathic Pain–Like Features in Chronic Musculoskeletal Pain: Protocol for an Open-Label Pilot Feasibility Clinical Trial

**DOI:** 10.2196/78806

**Published:** 2025-11-04

**Authors:** Luke Spencer Bialostocki, Divya Bharatkumar Adhia, Damith Rathnayake Mudiyanselage, Mark Llewellyn Smith, Yusuf Ozgur Cakmak, Dirk De Ridder, Ramakrishnan Mani, Jerin Mathew

**Affiliations:** 1 Department of Anatomy Faculty of Biomedical Sciences University of Otago Dunedin New Zealand; 2 Department of Surgical Sciences Faculty of Medicine University of Otago Dunedin New Zealand; 3 Neurofeedback Services of New York New York, NY United States; 4 Centre for Health, Activity, and Rehabilitation Research School of Physiotherapy University of Otago Dunedin New Zealand

**Keywords:** brain-computer interface, brain oscillations, chronic pain, electroencephalography, musculoskeletal, neuromodulation

## Abstract

**Background:**

Neuropathic pain (NP) is characterized as pain arising from lesions of the somatosensory nervous system. However, NP-like features have been found in several chronic secondary musculoskeletal (MSK) pain conditions in the absence of detectable lesion or damage to the somatosensory pathways. Emerging evidence has demonstrated associations between NP-like symptoms and altered neural activity within brain regions implicated in sensory perception and affective-emotional processing of pain with consistent findings of abnormal activity in the right insula (RIns) cortex and dorsal anterior cingulate cortex (dACC). Electroencephalography neurofeedback (EEG-NF) is a brain-computer interface biofeedback technique that allows individuals to self-regulate the real-time cortical brain activities of the regions of interest.

**Objective:**

The primary objective of this study is to investigate the feasibility and safety of a novel EEG-NF intervention designed to simultaneously downtrain activity in the RIns and dACC in individuals with a chronic secondary MSK pain condition exhibiting NP-like features. In addition, this study will conduct secondary exploratory analyses to investigate EEG-derived neuronal changes and their associations with clinical and experimental pain outcomes following the EEG-NF training.

**Methods:**

We will design a single-arm, open-label, pilot-feasibility trial. We will recruit adults aged 35-75 years with a score of ≥19 using the PainDETECT questionnaire and an average pain score of ≥4 on the 11-point Numeric Pain Rating Scale over the last 3 months, with a minimum pain duration of 3 months, to receive active EEG-NF training. Participants will receive auditory feedback as a reward for achieving a predetermined activity threshold of the RIns and dACC. Primary outcomes will evaluate feasibility, acceptability, and safety using both self-reported questionnaires and monitoring data. Collected data will be summarized descriptively, with mean (SD) reported where appropriate. Secondary outcomes will include EEG parameters, self-reported measures, heart rate variability, and quantitative sensory testing. An exploratory within-group pre-post statistical comparison will be conducted for all secondary outcome measures, and correlation analysis will be performed to explore relationships between EEG measures, self-reported outcomes, heart rate variability, and quantitative sensory testing measures.

**Results:**

This study has received approval from the Health and Disability Ethics Committee and is registered with the Australian New Zealand Clinical Trials Registry. Participant recruitment began in April 2025 and is ongoing. As of October 2025, data collection has been completed, with a total of 5 participants enrolled, all of whom have completed the study to date. We expect to complete the study in March 2026. This study will generate data on feasibility, safety, acceptability, and preliminary data to inform a fully powered effectiveness clinical trial.

**Conclusions:**

The results and data generated will inform the design and sample size calculation for a fully powered randomized controlled trial aimed at evaluating the effectiveness of EEG-NF in targeting NP-like features in individuals with chronic MSK pain.

**Trial Registration:**

Australian New Zealand Clinical Trials Registry ACTRN12625000706471; https://www.anzctr.org.au/Trial/Registration/TrialReview.aspx?id=389568&isReview=true

**International Registered Report Identifier (IRRID):**

DERR1-10.2196/78806

## Introduction

Chronic musculoskeletal (MSK) pain is the leading cause of disability, affecting over 1.7 billion people globally [1]. In New Zealand, one-quarter of the population is affected by some form of MSK disorder, and one-fifth of the population lives with chronic pain [2,3]. A subgroup of chronic MSK pain exhibits neuropathic pain (NP)-like qualities, which include symptoms such as burning, shocking, shooting, numbness, and “pins and needles” [4-8]. According to the International Association for the Study of Pain, NP is caused by a lesion or disease affecting the somatosensory system and may involve either the central or peripheral nervous system [9,10]. However, chronic secondary MSK conditions, such as knee osteoarthritis, low back pain, shoulder pain, neck pain, and osteoporosis, have been reported to exhibit NP-like qualities with widespread pain localization in 5.4%-33% cases in the absence of a verifiable lesion affecting the somatosensory system [4,8,11-14]. Similar findings have been observed in chronic widespread pain conditions, like fibromyalgia, which exhibit NP-like symptoms despite the absence of direct somatosensory lesions [15,16]. Moreover, patients with chronic MSK pain experience higher degrees of pain unpleasantness and bothersomeness, leading to emotional distress, which are part of the affective dimensions of pain [17,18]. Concurrently, the severity of symptoms or pain intensity may not always correlate to the extent of the MSK injury in these individuals [19,20].

Persistent pain associated with chronic MSK conditions and ongoing nociceptive input may lead to sensitization and widespread pain distribution, accompanied by altered sensory perception and changes in central processing within supraspinal structures [8,21]. Neuroimaging studies show that chronic MSK pain is associated with significant functional aberrations in brain regions involved in sensory processing, emotion and motivation, and pain inhibition [22,23]. Clinical aspects of pain correlate with these distinct anatomical brain regions and networks, as supported by functional neuroimaging data [24]. In particular, the insular cortex, specifically right insula (RIns) cortex and dorsal anterior cingulate cortex (dACC), plays a profound role in the sensory perception and emotional processing of pain and has been shown to exhibit alterations in chronic MSK pain conditions [25,26].

The insular cortex acts as a hub for cortical processing, including sensory, emotional, social, and cognitive information [27]. In particular, the insula serves as a hub linking multiple networks, including regions involved in pain mediation, such as the salience and ventral frontoparietal attention networks [28,29]. It has been shown that pain experience is associated with salience network, with the insular cortex serving as a key hub. The insular cortex is lateralized in function, with the RIns being predominantly involved in pain perception compared with the left insula [25]. Increased activation of the RIns has been reported in individuals with chronic MSK pain in both functional magnetic resonance imaging studies and electroencephalogram (EEG) studies in the gamma frequency band [30-32]. The insular cortex is also involved in modulating pain intensity, as it has been linked to pain catastrophizing [27,33]. Studies suggest that both the insula and the dACC are involved in the emotional processing of pain [34-37]. The dACC is the main proxy for the medial suffering pain pathway and is associated with motivational-affective component of pain [38]. Alterations in the activity of the dACC, such as increased activity in the alpha and beta frequencies and decreased functional connectivity to other regions, including the pregenual anterior cingulate cortex and somatosensory cortex, have been shown in people with chronic NP [38-40]. Previous studies have demonstrated that NP-like symptoms in individuals with painful knee osteoarthritis are associated with altered infraslow frequency (ISF) activity in the RIns and dACC, with significant correlations observed between ISF fluctuations and pain measures [30,41-44]. Therefore, targeting the ISF activity of both the RIns and dACC, which are associated with sensory and affective dimensions of pain, may modulate NP-like symptoms and produce clinical improvements in individuals with chronic MSK pain [26,27,38].

Currently, pharmacological treatments for NP conditions produce modest efficacy, potentially due to the variation in underlying mechanisms [45]. Furthermore, many available nonpharmacological treatments for NP conditions have demonstrated either low certainty of evidence or inconclusive results, highlighting the need for novel, innovative interventions [45,46]. Electroencephalography neurofeedback (EEG-NF) is a brain-computer interface biofeedback technique that allows individuals to self-regulate the real-time cortical brain activities of the regions of interest and reinforces learning using operant conditioning [47,48]. EEG-NF has been used successfully in several conditions, including chronic MSK pain, demonstrating clinical and EEG modulation [49-52]. EEG-NF training, which targets the ISF band in the brain, has been shown to provide clinical benefits [41,52]. Moreover, the ISF band has been shown to modulate higher-frequency brain oscillations through phase-locking mechanisms [53]. Previous studies have demonstrated that ISF EEG-NF training can influence resting-state brain networks and oscillations in various conditions, including targeting the dACC for chronic MSK pain [43,49,50,52,54,55]. However, to date, no studies have investigated the potential of ISF EEG-NF training for NP-like qualities in chronic MSK conditions. Furthermore, no research has explored the use of neurofeedback to simultaneously downtrain 2 distinct brain regions (dACC and RIns) as a targeted intervention for chronic MSK pain. This dual-target approach and ISF modulation for NP management highlight a novel strategy with the potential to achieve more comprehensive modulation of pain-related neural networks and clinical pain improvements.

Therefore, this study aims, for the first time, to explore the potential of downregulating the ISF activity of both the RIns and dACC using a novel ISF EEG-NF protocol in individuals with NP-like symptoms associated with chronic MSK pain conditions. Given the novelty of the proposed ISF EEG-NF training protocol, pilot testing of the protocol and assessment of feasibility and safety are necessary. The primary objectives of this study are (1) to pilot test and investigate the feasibility of the EEG-NF program, and (2) to investigate the safety of the ISF EEG-NF training. The secondary objectives are (1) to estimate the variability of the outcome measures for informing the sample size of a fully powered clinical trial, (2) to explore the immediate and short-term trends of the effects of the targeted ISF EEG-NF training on pain measures, and (3) to explore the changes in EEG current density activity at the targeted cortical regions (dACC and RIns) and the functional connectivity between these regions following ISF EEG-NF training.

## Methods

### Ethical Considerations

We obtained Ethical approval from the Health & Disability Ethics Committee, New Zealand (Reference 2023 EXP 19190). Research consultation with Māori was obtained from the Ngāi Tahu Research Consultation Committee (Reference 24402). The trial has been prospectively registered in the Australian New Zealand Clinical Trials Registry (ACTRN12625000706471).

All eligible participants will receive an information sheet with consent form outlining the study design and purpose. Participants who are enrolled will complete a digital consent form on the day before their baseline assessment and provide a signed paper copy upon attending in person. Participants may opt-out at any point during the study. All data for primary and secondary outcomes will be deidentified before analysis. In recognition of their time and contribution to the research, participants will receive NZ $200 (US $115) supermarket vouchers upon completion of all study sessions.

### Study Design

**Figure 1:**
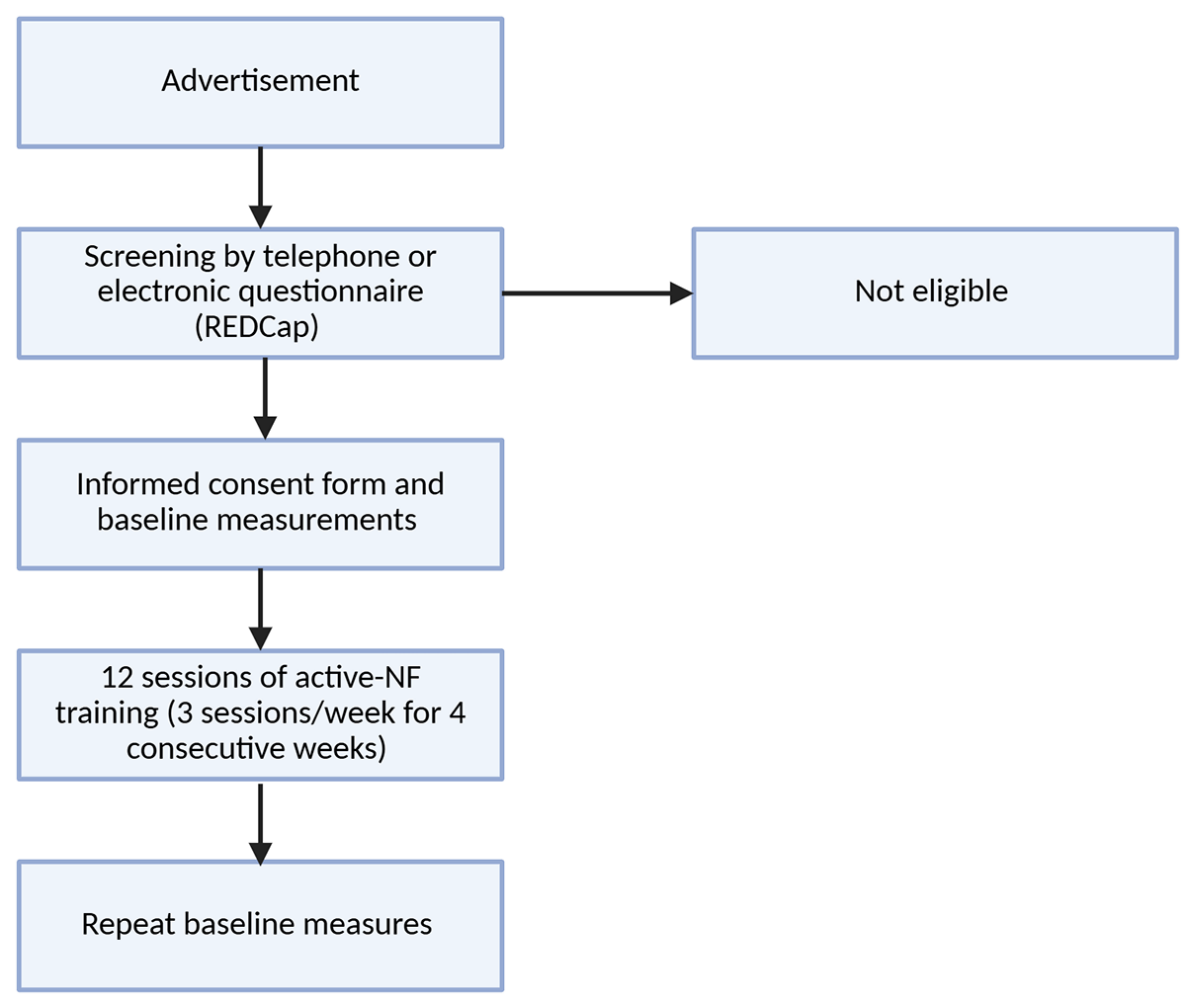
Diagram of participant flow for the study. NF: neurofeedback.

A single-arm, open-label, pilot-feasibility clinical trial was designed according to the Standard Protocol Items: Recommendations for Interventional Trials (SPIRIT; Multimedia Appendix 1) and the Consolidated Standards of Reporting Trials (CONSORT; Multimedia Appendix 2) extension for pilot and feasibility trials. The trial will be conducted in Dunedin, New Zealand, and the study phases are summarized in Figure 1 [56,57]. This study was designed as an open-label trial to assess the feasibility, acceptability, safety, and preliminary data of the developed EEG-NF training. Given the pilot-feasibility design and exploratory nature of the study, inclusion of a control group was not necessary at this stage. The primary focus is to gather preliminary data on EEG-NF training implementation and variability in clinical outcomes, which will inform a future fully powered clinical trial [58-60]. A structured description of the intervention is summarized in Multimedia Appendix 3, following the Template for Intervention Description and Replication (TiDiR) guide (Multimedia Appendix 3) [61].

### Recruitment

Convenience sampling will be used to recruit participants from the wider Dunedin community. Periodic advertising in community newspapers and social media will be carried out. Posters will be placed throughout the local community to advertise the study for participant recruitment.

Interested participants will undergo eligibility screening by telephone or through a secure online survey using Research Electronic Data Capture (REDCap; Vanderbilt University) web application [62]. As this is a pilot-feasibility study, a formal power-based sample size calculation was not undertaken. Instead, the sample size was determined as 6-12 participants over a 4-month recruitment period, in accordance with recommendations for feasibility studies and previous literature, ensuring adequacy to evaluate safety and feasibility outcomes while also permitting exploratory analyses [63].

### Eligibility Criteria

Participants aged 35-75 years with MSK pain, averaging a score of ≥4 on the 11-point Numeric Pain Rating Scale over the past 3 months, with a minimum pain duration of 3 months, and a score of at least 19 on the PainDETECT questionnaire will be eligible to participate in this study [6,14,64-67].

Participants will be excluded if they have one of the following conditions or situations**:** systemic rheumatic conditions, neurological conditions, major psychiatric illness, cervical or lumbar radiculopathy, peripheral entrapment neuropathies, cognitive impairment, have undergone any surgery or intra-articular steroid injections in the 3 months before the study, have undergone any intra-articular hyaluronic acid injections in the 6 months before the study, are scheduled to undergo any surgery within 6 months of enrollment in the study, have undergone previous neurological procedures of the brain, are pregnant or up to 6 months postpartum, cardiovascular disease, peripheral arterial disease of limbs, uncontrolled hypertension (≥150/95 mm Hg), or uncontrolled diabetes.

### Screening and Enrollment

Interested participants will undergo a brief preliminary screening for initial eligibility by telephone or through an online questionnaire using the REDCap online survey platform [68]. If the initial eligibility criteria are satisfied, participants will undergo a detailed screening based on their health information through a secure online survey. Participants will then be given an appointment for the final confirmatory screening.

A paper-based Montreal Cognitive Assessment will be carried out to screen for cognitive impairments [69]. The maximum Montreal Cognitive Assessment score is 30 points, and only those who score 21 or above will be eligible to participate in this study, based on normative data for the population included in the study [69,70]. If the participant meets this threshold and is found to be eligible, baseline assessment will continue.

Eligible participants will undergo 12 sessions (30 minutes each; 3 sessions per week) of ISF EEG-NF training and two 90-minute baseline and postintervention assessments at the Department of Anatomy–Research Clinic facility at the University of Otago, New Zealand. The intervention will be provided by a researcher experienced in the delivery of neurofeedback. Participants will be required to abstain from alcohol and caffeinated beverages for 24 hours and from food and drinks for at least 1 hour before any assessment sessions [71].

### Intervention: ISF EEG-NF Training

At the start of each session, participants will be seated in a chair with back support and asked to remain relaxed for 10 minutes while the trainer prepares them for neurofeedback training. The ISF EEG-NF training will be administered using a 21-channel DC-coupled amplifier (BrainMaster Technologies Inc) with a Comby EEG lead cap containing Ag/AgCl sensors of an appropriate size, secured to the participant’s head. Reference electrodes will be placed at the mastoids [72]. The impedance of the active electrodes will be continuously monitored to remain below 5 kΩ [49,52,72]. Participants will be advised to minimize eyeball, head, and neck movements, swallowing, and clenching of teeth to reduce motion artifacts in the EEG data [73]. Treatment adherence will be ensured by directly observing participants throughout the training sessions.

**Figure 2:**
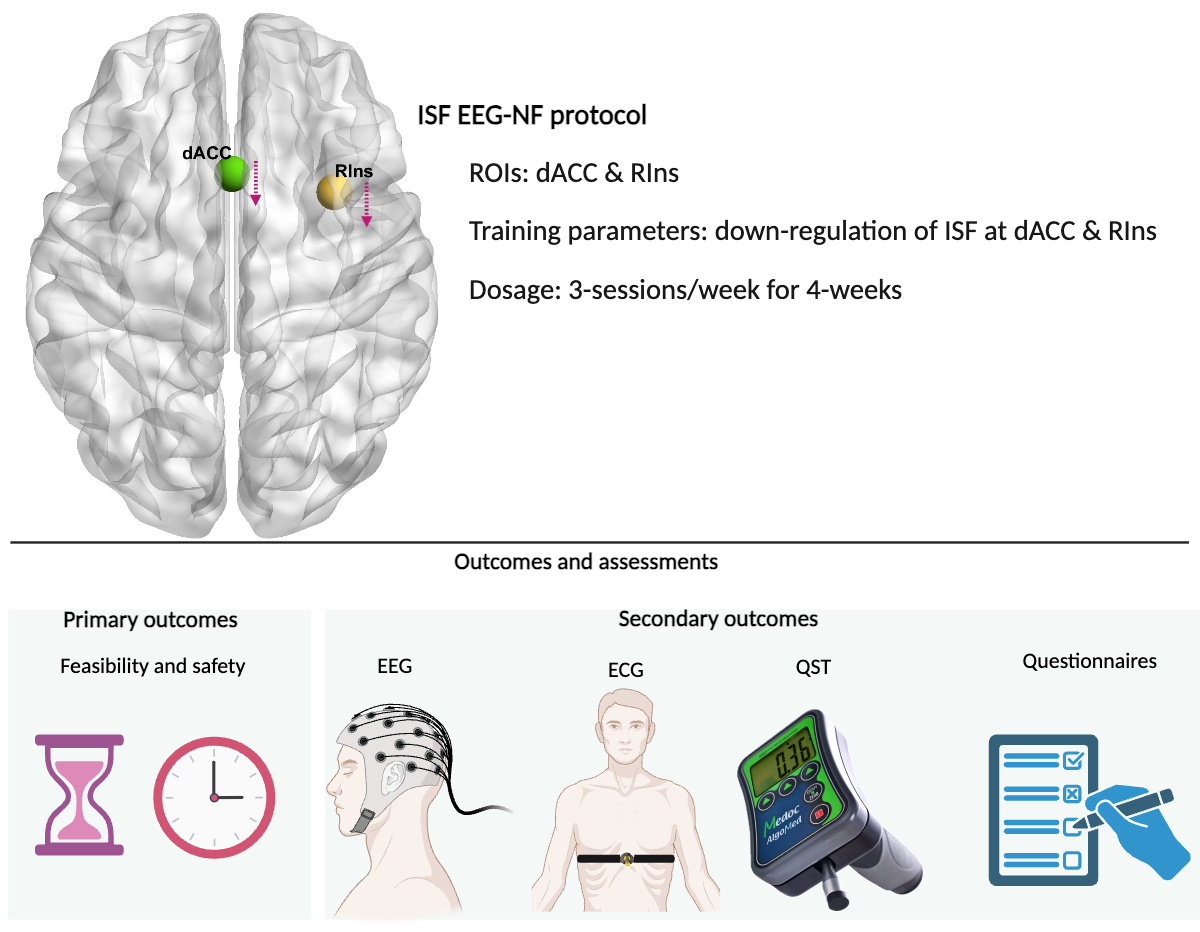
Regions of interest and outcome measures. dACC: dorsal anterior cingulate cortex; ECG: electrocardiogram; EEG-NF: electroencephalography neurofeedback; ISF: infraslow frequency; QST: quantitative sensory testing; RIns: right insula; ROI: region of interest.

Participants will be instructed to relax, keep their eyes closed, and listen to the audio feedback. For this study, an ISF EEG-NF program to downtrain the RIns and dACC was developed using the BrainAvatar Live standardized low-resolution electromagnetic brain tomography projector software [52,74]. Standardized low-resolution electromagnetic brain tomography allows the selection of brain regions (region of interest [ROI]) for EEG-NF training based on the current density of the ROI and 3D brain mapping functionality [74-76]. A visual representation of the ROIs, direction of training, and an overview of outcome measures are provided in Figure 2. The figure was generated using BrainNet Viewer [77] and BioRender [78].

The BrainMaster Technologies software (BrainAvatar) will deliver real-time auditory feedback when participants’ brain activity is simultaneously downregulated within the ROIs—the dACC and RIns—in the ISF (0.0-0.1 Hz) band. The BrainAvatar auditory feedback is generated using Musical Instrument Digital Interface–based tones and sound effects. The auditory feedback serves as a primary reinforcement modality, providing immediate and intuitive cues that reflect real-time brain activity. The system incorporates a range of sounds, including simple tones, chimes, and naturalistic effects such as chirps or gongs, which vary in pitch, clarity, or occurrence depending on whether the individual’s brainwave activity meets the targeted thresholds. For example, reward tones or sound effects are triggered only when the desired neural patterns are achieved, whereas the absence, distortion, or reduction of sound indicates deviation from the training goals. This dynamic modulation allows the brain to receive continuous, nonverbal information about its own functioning, supporting operant conditioning and the self-regulation processes central to effective neurofeedback training [47,48].

The auditory feedback (reward) will be captured using Audacity, a free and open-source audio recording and editing software [79]. Audacity can use the computer’s microphone and sound card as its own audio-to-digital converter, eliminating the need for additional equipment. The auditory feedback will then be used to calculate the duration of successful neurofeedback training, defined as the cumulative time that the participant received the auditory reward feedback during the 30-minute neurofeedback session [80]. The duration of successful neurofeedback training from each participant across the 12 sessions will be included in the analysis.

The target ROIs were chosen based on previous literature related to EEG-NF and MSK pain conditions [30,31,50,52]. The reward threshold will be continuously monitored by the trainer and adjusted manually if cortical activity falls outside the desired threshold [49,52,72]. The duration and number of neurofeedback sessions were informed by recent studies using ISF EEG-NF for chronic pain management, which demonstrated changes in clinical pain outcomes, and were selected to ensure methodological rigor in this study [72,81].

### Baseline Assessment

Once written informed consent is obtained, participants will complete questionnaires that include demographics and general health information, such as age, sex, and ethnicity. Assessment of resting-state EEG and clinical and experimental pain outcomes will be conducted by a researcher who is adequately trained in all procedures.

### Primary Outcomes

The primary outcomes of this study will be the feasibility and safety measures [82]. These will be collected by the investigator throughout the study period, from recruitment until the last postintervention assessment session. Feasibility outcomes include the recruitment rate, defined as the number of participants attending a screening assessment each month; the enrollment rate, defined as the proportion of participants recruited from the total screened; the compliance rate, defined as the number of sessions attended by each participant out of the total sessions; and the dropout rate, defined as the number of participants who dropped out over course of the study [83,84]. All measures will be monitored through monthly audits of recruitment records during the first 4 months of the recruitment and intervention period.

An adverse event is defined as any harmful event or symptom from the trial that could reasonably be linked to the procedure or EEG-NF training. EEG-NF is a safe technique; however, all participants will be asked about any adverse events experienced from the previous session at each visit, and any worsened side effects compared with previous training will be recorded. All participants will be instructed to complete the Discontinuation Emergent Sign and Symptom scale [85], a 43-item checklist consisting of emotional, behavioral, cognitive, and physical symptoms. These symptoms can be considered possible side effects from neurofeedback training and have been used in previous literature as a measure of safety monitoring for similar studies [52,72].

### Secondary Outcomes

Secondary outcomes of the study include self-reported questionnaires, resting-state EEG, electrocardiogram (ECG), and quantitative sensory testing (QST), assessed at baseline (T1) and postintervention (T2). Table 1 briefly describes each of the measurement tools used and the time points throughout the study. All tools are reliable and validated for use in individuals with persistent pain and are consistent with previous literature and the Initiative on Methods, Measurement, and Pain Assessment in Clinical Trials (IMMPACT) recommendations for clinical trials in chronic pain [86-90].

**Table 1:** Summary of secondary outcome measures, including descriptions and timeline used.

Outcome domain and constructs		Brief description of measurement tools		Measurement time points
**Pain**				
	Pain severity and interference [91,92]	The Brief Pain Inventory (BPI)–Short Form will be used to measure pain severity (0=no pain, 10=worst pain possible) and interference (0=does not interfere, 10=completely interfere) over the past 24 hours, past week, and past 4 weeks at T1 and T2. At each training session, a single-item BPI will measure pain severity and interference over the past 24 hours.	Full BPI: T1 and T2; Single-item BPI: every training session	
	Pain unpleasantness [93]	Measured using an 11-point numeric rating scale from 0 (not at all unpleasant) to 10 (most unpleasant imaginable) for the past 24 hours and past week at T1 and T2. At each training session, unpleasantness over the past 24 hours will be measured.	Before every session	
	Pain bothersomeness [41,94]	Measured using a 5-point scale ranging from “not at all” to “extremely” over the past 24 hours and past week at T1 and T2. Before every training session, bothersomeness over the past 24 hours will also be measured.	Before every session	
	Pain quality [6,8,66,95-100]	PainDETECT questionnaire: 12 items, each symptom rated on a 5-point scale. Scores <12 indicate nociceptive pain, 13-18 indicate possible neuropathic pain, and ≥19 indicate likely neuropathic pain. Short-form McGill Pain Questionnaire: 22 items, each rated on a 10-point numeric scale to assess pain quality. Leeds Assessment of Neuropathic Symptoms: 5 items with weighted scores ranging from 0 to 24, assessing signs of neuropathic pain. Dolour Neuropathique 4 interview: assess the presence of neuropathic pain symptoms; a cut-off score of 4 or more positive items indicates neuropathic pain.	T_1_ and T_2_	
	Fatigue [101]	Measured using a 10-point numeric rating scale ranging from 0 (no fatigue) to 10 (fatigue as bad as it can be).	T_1_ and T_2_	
**General health and well-being**				
	Depression, anxiety, and stress [102,103]	The Depression, Anxiety, and Stress Scale, containing 21 items. Each item is rated on a scale from 0 (never) to 3 (almost always) to quantitatively measure psychological distress.	T_1_ and T_2_	
	Pain personification [104]	Pain personification questionnaire, containing 11 items scored on a 5-point Likert scale (1=strongly disagree, 5=strongly agree) to measure the participant’s experience of pain as an internal object.	T_1_	
	Intolerance of uncertainty [105,106]	Intolerance of Uncertainty Scale, containing 12 items. Each item is rated on a 5-point Likert scale ranging from 1 (strongly disagree) to 5 (strongly agree).	T_1_	
	Sleep quality and disturbances [107,108]	Pittsburgh Sleep Quality Index, containing 19 items measuring sleep quality and disturbances over a 1-month interval. Total scores range from 0 to 21, with higher scores indicating poorer sleep quality.	T_1_ and T_2_	
**Participant perceptions about intervention and its impact on health and pain**				
	Credibility/expectancy of the intervention [109]	Credibility/Expectancy Questionnaire, containing 6 items to quantify how credible the participant perceived the intervention to be.	T_2_	
	Acceptability of interventions [110]	Numeric rating scale (0-7) assessing acceptability across 7 components of the theoretical framework of acceptability: affective attitude, burden, perceived effectiveness, ethicality, intervention coherence, opportunity costs, and self-efficacy.	T_2_	
	Perceived treatment satisfaction [111]	Single item numeric scale ranging from 0 (not satisfied at all) to 10 (highly satisfied).	T_2_	
	Patient Global impression of change [112,113]	The Patient Global Impression of Change score represents the participant’s perceived change in pain compared with baseline. It is rated on a 7-point scale, from 1 (very much improved) to 7 (very much worse).	T_2_	
	Mental fatigue [114]	Mental fatigue will be assessed using a single-item numeric scale from 0 (I do not feel fatigued at all) to 10 (I feel extremely fatigued).	T_2_	
	Mental strategies [72]	Patients will be asked whether they used any mental strategies during training sessions and if yes, to report which strategies they used.	T_2_	
	Level of motivation [52]	The Current Motivation–Brain Computer Interface will be used at T1. It uses a 7-point Likert scale across 4 components: mastery confidence, fear of incompetence, interest, and challenge related to electroencephalography neurofeedback training. A single-item 11-point numeric scale will be used for motivation before each training session, ranging from 0 (not motivated at all) to 10 (extremely motivated).	T_1_ and before every training session	
	Mood [52,115]	Mood will be measured before every neurofeedback session using a single item from the Brief Mood Introspection Scale, ranging from 0 (very unpleasant) to 10 (very pleasant).	Before every training session	
	Level of engagement with the training [52]	A 10-point numeric scale will be used, ranging from 1 (least engaged) to 10 (highly engaged).	After every training session	

### Electroencephalographic Measures

Resting-state EEG will be recorded (sampled at 256 Hz) using a 21-channel system (Fp1, Fp2, F7, F3, Fz, F4, F8, T7, C3, Cz, C4, T8, P7, P3, Pz, P4, P8, O1, O2, A1, A2; BrainMaster Technologies, Inc) [43]. Raw EEG recordings will be collected for 10 minutes with participants’ eyes closed. To minimize potential artifacts, participants will be instructed to avoid facial movements, head and neck movements, and swallowing. Alertness will be monitored by observing the presence of spindles and slowing of the alpha rhythm in the EEG stream to prevent drowsiness-related increases in theta power during the recording [116].

Each EEG file will be resampled to 128 Hz and bandpass filtered for 0.01-44 Hz in EEGLAB (MATLAB R2020a) [117]. Artifacts such as eye blinks, muscle artifacts, perspiration, and body movements will be filtered out using ICoN software [52,118].

Exact low-resolution brain electromagnetic tomography (eLORETA) software will be used to extract the following EEG variables. First, ROI current density, for which frequency-specific data will be collected by performing a voxel-by-voxel analysis containing 6239 voxels for each of the following frequency bands at the dACC and RIns: infraslow (0.01-0.10 Hz), slow (0.2-1.5 Hz), delta (2-3.5 Hz), theta (4-7.5 Hz), alpha (8-12 Hz), beta (12.5-30 Hz), and gamma (30.5-44 Hz). Comparisons will be made between participants’ pretreatment and posttreatment measures using eLORETA statistical contrast maps. Multiple voxel-by-voxel comparisons in the logarithm of t-ratio with a threshold of *P*≤.05 will be used to compute the cortical 3D distribution of current density [49].

Second, functional connectivity will be assessed using lagged linear connectivity, which is a statistical measure of coherence representing the phase synchronization between ROIs within the brain, as it combines the relationship between the phase and amplitude of the signals [23,24]. This measure reflects the level of communication between the ROIs [90]. Log-transformed lagged linear connectivity will be derived for all possible connections across the 7 frequency bands between the dACC and RIns.

eLORETA is an EEG source localization software that computes current density across the full brain volume [119] to localize cortical brain structures with fewer localization errors for EEG data extraction and analysis [120]. eLORETA has been widely used in previous literature for EEG analysis [121-123].

### Cardiovascular Measures

Heart rate variability (HRV) metrics will be obtained using a Polar V800 heart rate monitor and a Polar H10 Chest Pro Strap [124]. The ECG monitor will be placed below the sternum and secured to ensure a fixed position for the participant. Raw heart rate and R-R (peak-to-peak R wave) interval time series data will be downloaded from the Polar Flow software for further processing and analysis. Kubios HRV Premium (v3.2.0) software will be used to analyze HRV from the R-R interval record exported from the Polar Flow software [125]. The root-mean-square of successive differences between normal heartbeats and stress index (SI) will be extracted for HRV analysis. Root-mean-square of successive differences quantifies the variability between adjacent respiratory rate intervals, demonstrating a lesser susceptibility to influence by the respiratory system and, due to this, is regarded as the most dependable HRV for monitoring vagal response [125,126]. The SI will be calculated using the square root of the Baevsky SI model [127]. Given that the insular cortex and dACC are key regions involved in interoception—integrating internal bodily signals to regulate physiological and emotional states—any changes in sympathetic and parasympathetic activity can be effectively captured through HRV analysis [80,128,129].

### Quantitative Sensory Testing Measures

The following QST measurements will be performed at T1 and T2. All QST measures were included based on previous literature demonstrating variability in these clinical measures among individuals with chronic MSK pain and NP [130-132].

#### Pressure Pain Threshold

A computerized algometer (AlgoMed, Medoc) will be used to measure pressure pain threshold on the dorsal aspect of the nondominant wrist of the participant for 3 trials [133,134]. Two familiarization trials will be performed at the mid-forearm before the formal trials. Pressure will be applied to the test site gradually at a rate of 30 kPa/s for each participant by placing a 1-cm^2^ probe perpendicular to the site until the participant reports that the pressure has become a pain sensation. Participants will be instructed to verbally inform the investigator immediately when they perceive pressure as pain. The average of the 3 tests will be used for analysis [135].

#### Punctate Pain Intensity

Punctate pain intensity will be assessed using a nylon filament (Semmens monofilament 6.65, 300 g) on the nondominant wrist of the participant for 3 trials [134,136]. The nylon monofilament will be applied perpendicular to the site on the nondominant wrist with enough force to bend the filament. Immediately after each trial, participants will report their pain intensity on a 101-point numeric pain rating scale (0 indicating no pain at all and 100 indicating the most intense pain they have ever experienced) [137]. The average across the 3 trials will be calculated.

#### Mechanical Temporal Summation

Mechanical temporal summation (MTS) will be assessed using a nylon filament (Semmens monofilament 6.65, 300 g) on the dorsal aspect of the nondominant wrist of the participant for 3 trials [134]. Participants will be instructed to provide a pain rating on a 101-point numeric pain rating scale. Immediately after this, 10 consecutive contacts with an interstimulus interval of 1 second will be performed, and participants will be asked to provide a pain rating on the 101-point numeric pain rating scale again. MTS will be calculated as the difference between the initial value after the first contact and the highest pain rating after the tenth contact. The average of these 3 trials will then be used in analysis.

#### Vibration Detection Threshold

A tuning fork (64 Hz, 8/8 scale) will be used to test a participant’s ability to detect vibration and will be placed on the dorsal aspect of the nondominant wrist with suprathreshold vibration intensity. It will be held in place until the participant can no longer feel the vibration [134,138,139]. This will be graded on an 8/8 scale, with 8 indicating the highest level of sensitivity to vibration [140]. The vibration detection threshold will be determined using the mean of 3 consecutive tests for each location [141].

#### Medication and Other Treatment Monitoring

Any concurrent treatments will be monitored, including regular general practitioner visits, use of physiotherapists, osteopaths, chiropractors, and self-management as well as current medications. Any medications that participants are taking regularly will be recorded at the baseline assessment. Changes in dosage, as well as the frequency of use of pain-relief medications during the study period, will be noted at each session.

### Analysis

#### Primary Outcomes

Participant demographics, feasibility, acceptability, credibility, and safety data over the course of the study will be summarized descriptively [142]. Mean (SD) or median (IQR) when distributional assumptions are not met and CI (75% and 95%) of the clinical and quantitative outcome measures for the participants will be derived using GraphPad Prism software [143]. Feasibility will be assessed against prespecified progression criteria: ≥50% of eligible participants consenting, recruitment meeting target within the planned window (~6 participants in 4 months), retention ≥80% at postintervention, adherence ≥75% of scheduled sessions, data completeness ≥85%, and mean acceptability ≥70%. Each metric will be reported as a point estimate with 95% CIs, and progression will be discussed in light of these thresholds [60,144,145].

#### Secondary Outcomes

A within-group pre-post statistical comparison (to be determined based on the normality of the data) will be conducted for all the secondary outcome measures using GraphPad Prism software (version 10.4.1; GraphPad Software) [143]. Similarly, correlation analysis will be performed to explore the relationship between EEG measures, self-reported outcomes, and HRV and QST measures. All analyses for this pilot-feasibility study will be unpowered due to sample size and are thus exploratory in nature.

Secondary outcomes will be interpreted descriptively and exploratorily, focusing on patterns, effect size estimates, and CIs rather than formal hypothesis testing, to generate preliminary trends and inform the design of a future powered clinical trial [146]. Changes in clinical ratings before and after the intervention will be analyzed to inform the design of future efficacy trials, using effect sizes, paired *t* tests, and Wilcoxon signed-rank tests.

## Results

This study was funded in January 2025. As of October 2025, data collection has been completed, with a total of 5 participants enrolled. The target sample size for the study was 12 participants. Data analysis will commence in November 2025 and will continue for 3 months following the completion of data collection. The results of the study are expected to be published once data collection and analysis are complete in late 2026 or early 2027. Any deviations from the protocol, regardless of the reason, will be documented in the Australian New Zealand Clinical Trials Registry and reported in the final publication.

## Discussion

### Principal Results

The primary aim of this study is to pilot test and assess the feasibility of the novel ISF EEG-NF training protocol targeting the dACC and RIns for individuals with NP-like qualities. This study, for the first time, aims to simultaneously downregulate the ISF activity of the RIns and dACC in a chronic pain population. Therefore, a preliminary study assessing the feasibility, acceptability, and safety of the intervention is imperative before advancing to a fully powered clinical trial to evaluate its efficacy.

### Comparison With Previous Work

Previous studies, using comparable protocols have conducted dual region source-localized ISF EEG-NF training in individuals with chronic low back pain and major depressive disorders [50,147]. These studies successfully targeted the dACC, showing clinical improvements and variability following the training. In addition, a recent study using a similar methodology successfully targeted the insular cortex, specifically training the left posterior insula in healthy volunteers. This study explored the relationship between the duration of successful neurofeedback training and clinical outcomes, aiming to understand the impact of EEG-NF training on gastric slow-wave activity [80]. Thus, this study adopts a similar methodological framework to develop the ISF EEG-NF program [75,120], using established and reliable methods that have been successfully implemented in previous research [43,52,72,80,89]. The ROIs (RIns and dACC) are created with all the nearby voxels with a localization of 5 mm [41,52,120]. Moreover, no studies have attempted ISF EEG-NF to manage NP-like qualities in individuals with chronic MSK pain, highlighting the novelty of this project and its potential for future clinical translation.

This study will also introduce a novel approach to measuring EEG learning effects by assessing the duration of successful neurofeedback within each session, a method previously used in only one study [80]. The secondary aims will allow for exploratory analysis to better understand data variability and trends in EEG measures and clinical pain outcomes in individuals with NP-like qualities in chronic MSK conditions.

### Limitations

During this study, several challenges may be encountered while assessing feasibility. One of the primary issues may be due to recruitment of participants. This could be attributed to the challenges faced by patients coming to the study location to receive treatment, as their pain may be debilitating, as highlighted in previous literature emphasizing activity limitations [148]. This study is also targeting a demographic that may be spread throughout the wider local community and thus may not encounter the flyers placed throughout the community. We plan to mitigate this by also including the study advertisement in newspapers, which may be more accessible for recruiting patients who are further away from the surrounding area. Since PainDETECT is being used as a screening tool, we are excluding patients with scores below 19, including those falling within the range of 14-18 that indicates a possible neuropathic component. This produces a challenge as it reduces recruitment and potentially excludes patients who may have a neuropathic component to their pain. However, only recruiting patients who score 19 or above strengthens the validity of the patients having a neuropathic component, as painDETECT has a high sensitivity and specificity [149]. That said, we acknowledge that this can impact patient recruitment and enrollment rates.

### Conclusions

The data and findings of this study will aid in designing future fully powered clinical trials to further evaluate the efficacy of this novel ISF EEG-NF training for NP management. Additionally, the foundational data from this pilot open-label feasibility trial will provide the necessary information to enable future studies to perform power calculations for designing upcoming trials.

## Appendix

Multimedia Appendix 1 SPIRIT checklist.

Multimedia Appendix 2 CONSORT extension for pilot and feasibility trials.

Multimedia Appendix 3 TiDiR checklist.

Multimedia Appendix 4 Peer review report from the International Association for the Study of Pain (IASP) Early Career Researcher Grant Review Committee.

## Abbreviations

CONSORT: Consolidated Standards of Reporting Trials
dACC: dorsal anterior cingulate cortex
ECG: electrocardiogram
EEG: electroencephalogram
EEG-NF: electroencephalography neurofeedback
eLORETA: exact low-resolution brain electromagnetic tomography
HRV: heart rate variability
IMMPACT: Initiative on Methods, Measurement, and Pain Assessment in Clinical Trials
ISF: infraslow frequency
MSK: musculoskeletal
MTS: mechanical temporal summation
NP: neuropathic pain
QST: quantitative sensory testing
REDCap: Research Electronic Data Capture
RIns: right insula
ROI: region of interest
R-R: peak-to-peak R wave
SI: stress index
SPIRIT: Standard Protocol Items: Recommendations for Interventional Trials
TiDiR: Template for Intervention Description and Replication

## Data Availability

The data generated during this study will be available on reasonable request from the corresponding author.
