# Supplementary material for: Neurofeedback Training for Managing Neuropathic Pain–Like Features in Chronic Musculoskeletal Pain: Protocol for an Open-Label Pilot Feasibility Clinical Trial
Source: JMIR Res Protoc. 2025 Nov 4;14:e78806. doi: 10.2196/78806 (PMC12627977; doi:10.2196/78806)
Supplement: Multimedia Appendix 3 [file resprot_v14i1e78806_app3.pdf]

TIDieR
Template for Intervention Description and Replication

The TIDieR (Template for Intervention Description and Replication) Checklist*:

Information to include when describing an intervention and the location of the information

Item number	Item	Where located ** Primary paper (page or appendix number)	Other † (details)
	**BRIEF NAME**		
1.	Neurofeedback training for neuropathic pain-like qualities	1	NA
	**WHY**		
2.	Research shows that individuals with chronic NP-like symptoms in MSK conditions exhibit heightened activity in cortical brain regions related to sensory perception (RIns) and emotion (dACC). Thus, this study aims to modulate the ISF oscillations in the RIns and dACC through EEG-NF. Previous studies have successfully reported the use of ISF EEG-NF for managing MSK pain.	3-5	NA
	**WHAT**		
3.	The ISF EEG-NF training program will be administered with a 21-channel DC-coupled amplifier from BrainMaster Technologies using an EEG gap with Ag/AgCl electrodes.	10	NA
4.	The Comby EEG lead cap with sensors (Ag/AgCl) of an appropriate size will be fixed secured to the participant's head with reference electrodes being placed at the mastoids. The impedance of the active electrodes will be checked to continuously remain below five kilo-ohms. Participants will be instructed to close their eyes, relax and listen to the sound being played prior to the commencement of training. A distinct tone will be played when the participant's brain activity meets reduced ISF magnitude at the dACC and RIns.	10-12	NA
	**WHO PROVIDED**		
5.	An investigator who is adequately trained to provide EEG-NF intervention.	10	NA

	**HOW**		
**6.**	Each participant will receive face to face ISF-EEG-NF training.	10-11	NA
	**WHERE**		
**7.**	Participants will receive the EEG-NF training at the Department of Anatomy-Research Clinic facility at the University of Otago, New Zealand.	10	NA
	**WHEN and HOW MUCH**		
**8.**	All participants will be required to attend 12 sessions (30-min each; three sessions per week; for four consecutive weeks) of training. Assessment of clinical and EEG outcomes will be carried out at two separate sessions of 90-min duration: baseline assessment and immediately following the final treatment session.	10	NA
	**TAILORING**		
**9.**	Intervention is personalised. All the participants will receive auditory feedback based on their real-time cortical activity recorded during each training session. If required, manual threshold adjustments will be done based on the real-time electrical activity of each participant, during each session.	12	NA
	**MODIFICATIONS**		
**10.‡**	NA	NA	NA
	**HOW WELL**		
**11.**	Intervention adherence will be maintained across each participant for every session. All the participants will undergo 12 sessions of NF training for 30 min. The NF program is default set for 30 min of training.	14	NA
**12.‡**	Intervention adherence, in relation to how many sessions the participant attended, will be reported in the feasibility measures after the post-intervention session.	14	NA

** **Authors** - use N/A if an item is not applicable for the intervention being described. **Reviewers** – use '?' if information about the element is not reported/not sufficiently reported.

† If the information is not provided in the primary paper, give details of where this information is available. This may include locations such as a published protocol or other published papers (provide citation details) or a website (provide the URL).

‡ If completing the TIDieR checklist for a protocol, these items are not relevant to the protocol and cannot be described until the study is complete.

* We strongly recommend using this checklist in conjunction with the TIDieR guide (see *BMJ* 2014;348:g1687) which contains an explanation and elaboration for each item.

* The focus of TIDieR is on reporting details of the intervention elements (and where relevant, comparison elements) of a study. Other elements and methodological features of studies are covered by other reporting statements and checklists and have not been duplicated as part of the TIDieR checklist. When a **randomised trial** is being reported, the TIDieR checklist should be used in conjunction with the CONSORT statement (see www.consort-statement.org) as an extension of **Item 5 of the CONSORT 2010 Statement.** When a **clinical trial protocol** is being reported, the TIDieR checklist should be used in conjunction with the SPIRIT statement as an extension of **Item 11 of the SPIRIT 2013 Statement** (see www.spirit-statement.org). For alternate study designs, TIDieR can be used in conjunction with the appropriate checklist for that study design (see www.equator-network.org).
