# Supplementary material for: Neurofeedback Training for Managing Neuropathic Pain–Like Features in Chronic Musculoskeletal Pain: Protocol for an Open-Label Pilot Feasibility Clinical Trial
Source: JMIR Res Protoc. 2025 Nov 4;14:e78806. doi: 10.2196/78806 (PMC12627977; doi:10.2196/78806)
Supplement: Multimedia Appendix 4 [file resprot_v14i1e78806_app4.pdf]

Title	**0001**	09/13/2024
	by **Jerin Mathew** in **2025 Early Career Research Grant**	id. 47493986
	Average score: n/a	

Reviewed by: (Score:)

11/02/2024

Applicant-professional standing, level of independence, research team involved	20
Impact and feasibility of the proposal	20
Strengths	I am not an expert in this topic area but the proposal seems to be well thought-out and the project seems feasible. The applicant and the proposed co-investigators have the relevant expertise to undertake the work. The timeline is clearly set out and the implications of the work and next steps for the project team are clearly reported.
Weaknesses	No specific comments.

Reviewed by: (Score:) 10/20/2024

Applicant-professional standing, level of independence, research team involved	20
Impact and feasibility of the proposal	16
Strengths	1. Dr. Mathew is an exceptionally promising early-career investigator who received his PhD in 2022 and is a physiotherapist with a solid foundation in neuroscience. He has a strong track record of productivity with 28 publications and several grants serving as principal or co-investigator. The candidate’s recommendation letters are very strong, and he has received institutional support to carry out the study.<br><br>2. The study aims to assess the feasibility and efficacy of neurofeedback (NF) for downregulating electrical activity in the insular and cingulate cortices in individuals with chronic musculoskeletal disorders and neuropathic symptoms. The cortical targets for NF training and the direction of electrical activity regulation were guided by the candidate’s prior research. The background section offers a compelling rationale for using EEG-based NF to address neuropathic symptoms in this population. This study has the potential to significantly enhance pain management, and the application is further strengthened by well-defined future research directions and a solid plan for advancing this work.<br><br>3. The study aims are supported by promising preliminary findings from the investigators, suggesting that neuropathic symptoms in knee osteoarthritis are associated with heightened activation in the insular and anterior cingulate cortices. These findings provide a solid foundation for the current study and its aim to investigate whether NF can downregulate these regions and alleviate neuropathic symptoms.

Weaknesses	1. Several critical aspects of the methodology lack sufficient detail and require further elaboration. Specifically, more information is needed on how NF trains participants to regulate the brain's electrical activity. Per the application, the intervention provides no explicit instructions regarding mental strategies to be used, yet participants receive sound feedback indicating their brain activity is responding to the training. How does this process work? Do participants observe a visual display during training sessions, or are they engaged in an activity (e.g., playing a computer game)? What instructions, if any, are given to guide their actions? Additionally, what happens if participants' brain activity fails to meet the selected threshold? Without these specifics, it is challenging to fully evaluate the potential significance and impact of the project.<br><br>2. The investigators propose 9 sessions for the training, each lasting 30 minutes. However, there is insufficient justification for why this duration and number of sessions would be enough to achieve meaningful treatment effects.<br><br>3. The participant age range is wide, yet there is no mention of whether or how age will be considered in the analytic approach, in addition to other potentially relevant characteristics (e.g,. sex, gender).<br><br>4. There are numerous measures to be administered (e.g., psychological health, general health, sleep), but it is unclear how these will be integrated into the analysis and which instruments will be used to assess these areas. Moreover, there is no specific detail about the quantitative sensory testing (QST) to be conducted, nor the rationale for its inclusion in relation to the study aims. Similarly, the inclusion of physical activity lacks justification, and it is unclear how it contributes to the study's objectives.
